# A case of early gastric cancer with metastatic recurrence following curative endoscopic submucosal dissection

**DOI:** 10.1002/deo2.326

**Published:** 2023-12-28

**Authors:** Yukihiro Morita, Hidenori Kimura, Osamu Inatomi, Akito Noguchi, Shuhei Shintani, Takayuki Imai, Masashi Ohno, Hiroshi Matsumoto, Atsushi Nishida, Sachiko Kaida, Masaji Tani, Ryoji Kushima, Akira Andoh

**Affiliations:** ^1^ Department of Medicine Shiga University of Medical Science Shiga Japan; ^2^ Department of Surgery Shiga University of Medical Science Shiga Japan; ^3^ Department of Pathology Shiga University of Medical Science Hospital Shiga Japan

**Keywords:** curative resection, early gastric cancer, endoscopic submucosal dissection, eCuraA, metastatic recurrence

## Abstract

A 70‐year‐old man was diagnosed with early gastric cancer with ulcerative findings. Endoscopic submucosal dissection as an absolute indication was performed, and en bloc resection was achieved. Pathological examination revealed a well‐differentiated adenocarcinoma, 3 × 2 mm in size, intramucosal, with an ulcerative scar, no lymphovascular invasion, and a tumor‐free margin. We diagnosed it as a curative resection and followed up with annual endoscopy. Sixteen months after endoscopic submucosal dissection, esophagogastroduodenoscopy revealed a singular ulcer scar; however, serum carcinoembryonic antigen level was elevated. Computed tomography scan showed wall thickening of the gastric antrum and an irregular mass on the dorsal side. Additionally, 18F‐fluorodeoxyglucose positron emission tomography/coomputed tomography showed 18F‐fluorodeoxyglucose uptake in the gastric antrum, irregular mass, and liver. Endoscopic ultrasonography revealed an internally heterogeneous mass in the gastric antrum region extending from the submucosal layer to the muscularis propria layer. Using an endoscopic ultrasonography‐guided fine needle biopsy with a 22‐gauge needle for the mass, we diagnosed local recurrence with the submucosal tumor‐like appearance, lymph node metastasis, and liver metastases. Unfortunately, the patient died of gastric cancer 3 months after the diagnosis. Here, we report a rare case of local recurrence in the submucosal layer, lymph node metastasis, and liver metastases 16 months after curative endoscopic submucosal dissection.

## INTRODUCTION

Endoscopic submucosal dissection (ESD) is now widely used as a minimally invasive treatment for early gastric cancer with a very low risk of lymph node metastasis.^1^ The absolute indication of ESD in the Japanese Gastric Cancer Treatment Guideline 2021 (6th edition) includes differentiated‐type mucosal adenocarcinoma measuring ≤ 3 cm with ulcerative findings (UL1). In the Japan Oncology Group 0607 study, 470 patients with early gastric cancer including 207 patients with UL1 (≤3 cm) were enrolled, and 317 patients who achieved curative endoscopic resection had no recurrence.^2^ However, we encountered a case of local recurrence in the submucosal layer, lymph node metastasis, and liver metastases 16 months after curative endoscopic submucosal dissection.

## CASE REPORT

**FIGURE 1:**
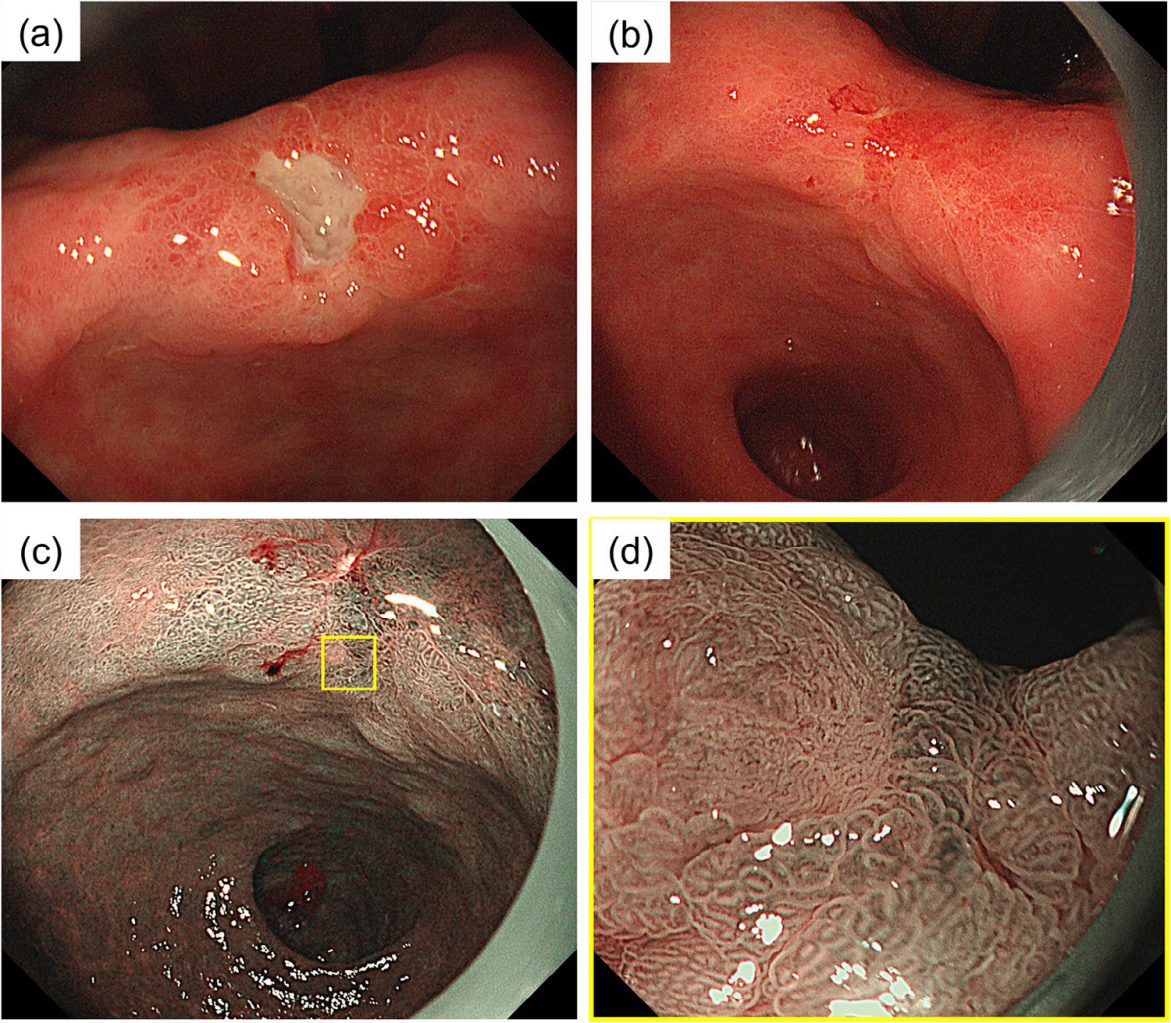
Esophagogastroduodenoscopy (EGD) showing early gastric cancer on the lesser curvature of the angulus. (a) EGD showing an irregular ulcerative lesion. (b) EGD 3 months later showing an ulcer scar. (c, d) The lesion showing an irregular microsurface pattern with a demarcation line on magnifying narrow‐band imaging.

**FIGURE 2:**
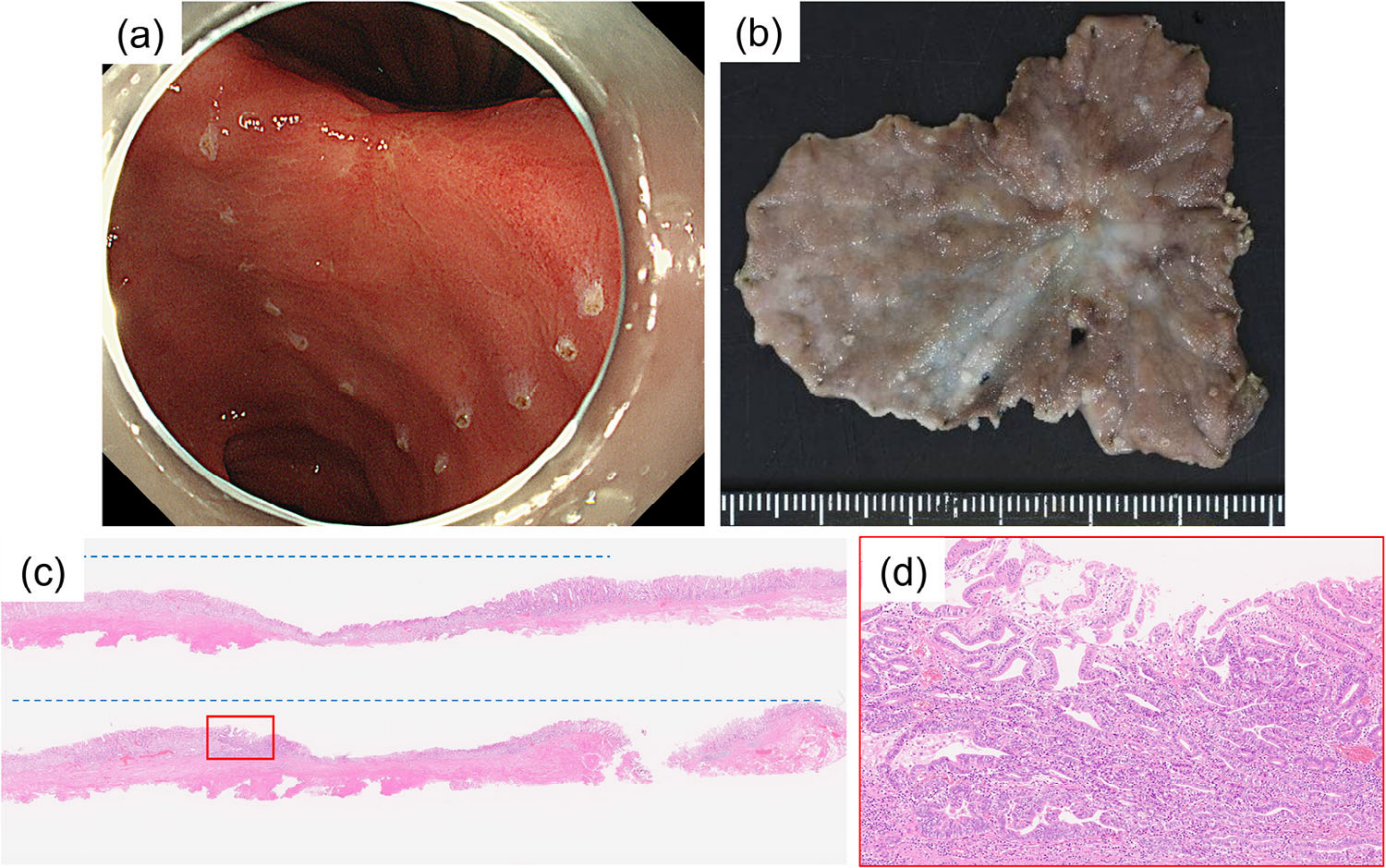
(a, b) Early gastric cancer resected en bloc by endoscopic submucosal dissection. (c, d) Pathological examination revealing well‐differentiated adenocarcinoma (tub1) of 3 × 2 mm in size (red box), intramucosal, fibrosis by an ulcer scar in the submucosal layer (blue broken line). No lymphovascular invasion and the margins are tumor‐free.

A 70‐year‐old man who underwent ESD for superficial esophageal squamous cell cancer 5 years ago underwent esophagogastroduodenoscopy (EGD) for surveillance. An irregular ulcerative lesion was detected on the lesser curvature of the angulus (Figure 1a). The biopsy specimen was difficult to diagnose as neoplastic or non‐neoplastic. Treatment with 20 mg of vonoprazan was continued and EGD 3 months later revealed that the ulcer had healed and an ulcer scar was present (Figure 1b). A small pale mucosa was observed on the anal side of the scar and magnifying narrow‐band imaging revealed an irregular microsurface pattern with a demarcation line (Figure 1c,d). The biopsy specimen revealed a well‐differentiated tubular adenocarcinoma. Enhanced computed tomography (CT) revealed neither lymph node nor distant metastasis. The clinical diagnosis was stage IA gastric adenocarcinoma (cT1aN0M0), and ESD was performed as an absolute indication (Figure 2a). Despite encountering challenges in resecting the lesion due to excessive submucosal fibrosis of the ulcer scar, and the procedure taking an extended duration (195 min), en bloc resection was achieved without any adverse events (Figure 2b). Pathological examination of the resected specimen revealed a well‐differentiated adenocarcinoma (tub1), 3 × 2 mm in size, intramucosal, with an ulcer scar, no lymphovascular invasion, and a tumor‐free margin (Figure 2c,d). Although cutting into the lesion from the cutting‐plane side was observed in a part of the resected specimen, this cutting did not extend into the tumor component; therefore, we diagnosed it as endoscopic curability A (eCuraA) according to the Japanese Gastric Cancer Treatment Guideline 2021 (6th edition).^3^ Three months after the ESD, the patient underwent an EGD and CT scan for examination of the black stool. As EGD revealed bleeding from the ulcer after ESD, endoscopic hemostasis was performed. CT indicated no evidence of recurrence, and 5 months after ESD, EGD showed no findings of recurrence, confirming ulcer healing. Subsequently, the patient underwent annual endoscopy.

**FIGURE 3:**
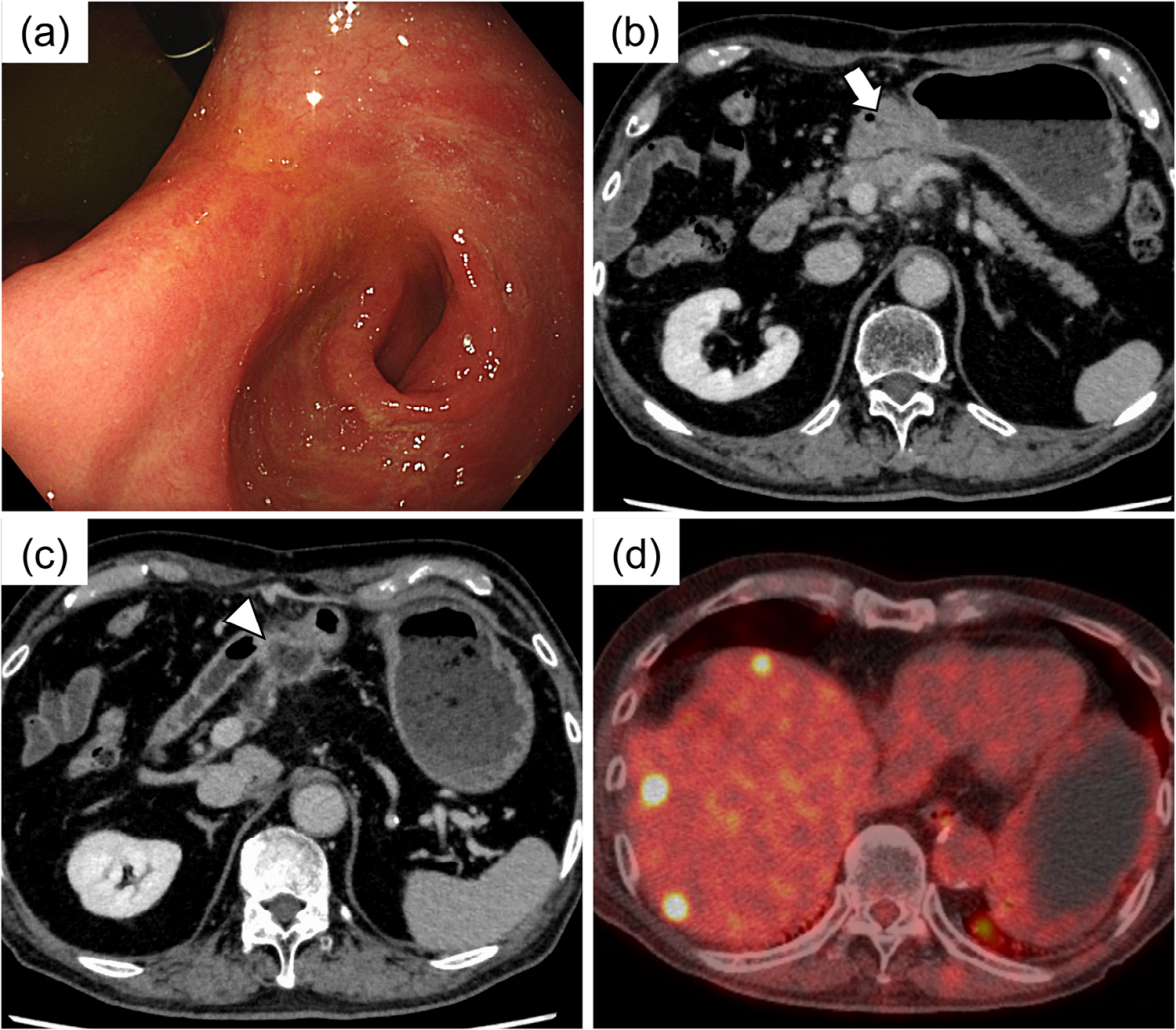
Endoscopic findings, computed tomography (CT), and 18F‐fluorodeoxyglucose positron emission tomography (FDG‐PET)/CT images. (a) Esophagogastroduodenoscopy (EGD) showing a singular ulcer scar. (b, c) CT revealing wall thickening of the gastric antrum (white arrow) and an irregular mass on its dorsal side (white arrowhead). (d) FDG‐PET/CT showing FDG uptake in the liver.

**FIGURE 4:**
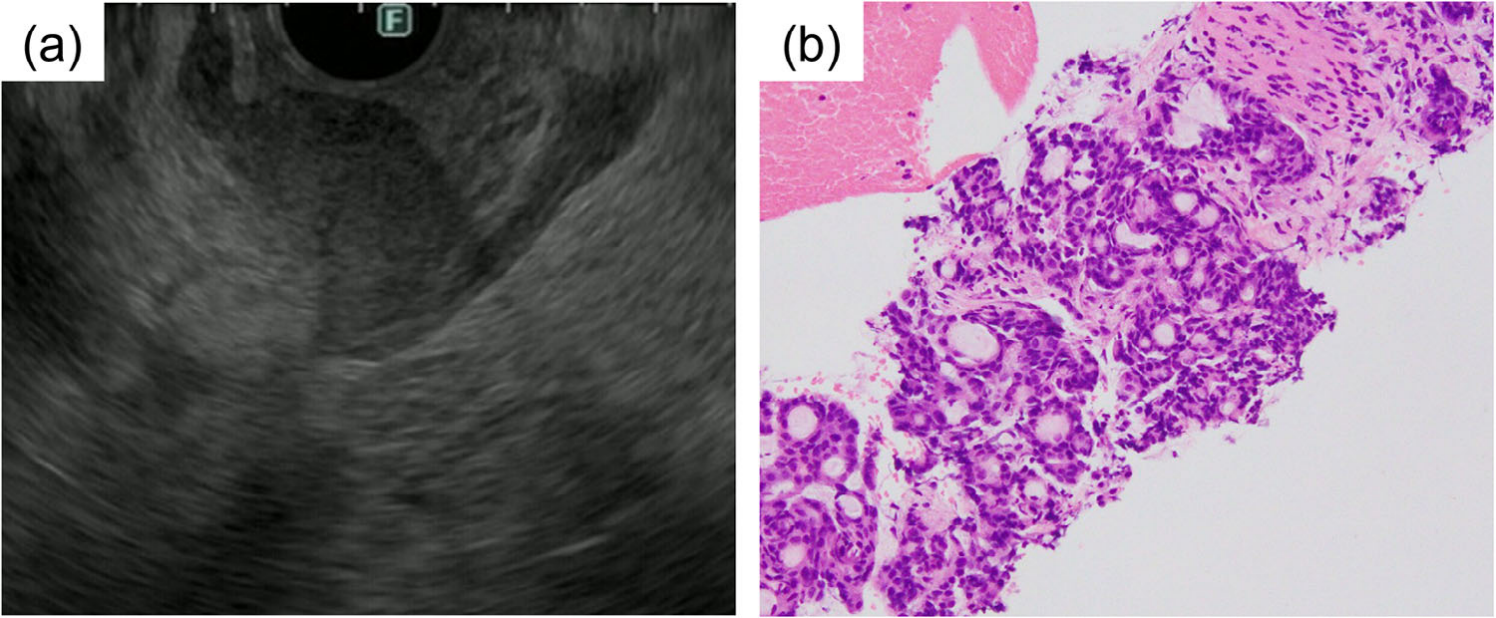
(a) Endoscopic ultrasonography showing an internally heterogeneous mass in the gastric antrum region extending from the submucosal layer to the muscularis propria layer. (b) Endoscopic ultrasonography‐guided fine needle biopsy specimens showing tubular adenocarcinoma.

Sixteen months after the ESD, the patient underwent EGD for surveillance and blood analysis. Although EGD revealed only an ulcer scar (Figure 3a), the serum carcinoembryonic antigen level was elevated to 7.4 ng/mL (normal range < 5.0 ng/mL). Therefore, CT was performed, which revealed wall thickening of the gastric antrum and an irregular mass on the dorsal side (Figure 3b,c). In addition, 18F‐fluorodeoxyglucose positron emission tomography (FDG‐PET)/CT showed FDG uptake in the gastric antrum, irregular mass, and liver (Figure 3d). Endoscopic ultrasonography (EUS) showed an internally heterogeneous mass in the gastric antrum region extending from the submucosal layer to the muscularis propria layer (Figure 4a). As there were no other predominant findings in the imaging studies, we considered a possible recurrence of gastric cancer and performed an EUS‐guided fine needle biopsy with a 22‐gauge needle (EUS‐FNB). The EUS‐FNB specimen showed tubular adenocarcinoma (Figure 4b), and immunochemical staining was similar to that of the resected ESD specimen (CK7: +; CK20: +; CDX2: ‐; HNF4α: +; SATB2: ‐; p53: wild type; carcinoembryonic antigen: +). Thus, we diagnosed local recurrence with the submucosal tumor‐like appearance, lymph node metastasis, and liver metastases after curative resection. Palliative gastrojejunal bypass surgery was performed because of malignant stenosis caused by recurrent gastric cancer. Once the patient was able to consume food, his intake gradually declined, and he died of gastric cancer 3 months after the diagnosis.

## DISCUSSION

According to the current 6th edition of the Guidelines for Gastric Cancer Treatment, eCuraA is characterized by the fulfillment of the following criteria: en bloc resection, negative horizontal margin (HM0), negative vertical margin (VM0), no lymphovascular infiltration (Ly0, V0), differentiated type‐dominant adenocarcinoma, pT1a, UL0, any tumor size or UL1, ≤ 3 cm; and undifferentiated type‐dominant adenocarcinoma, pT1a, UL0, ≤ 2 cm. Follow‐up with annual endoscopy is recommended to detect metachronous gastric cancer after the eCuraA resection.^3^ In the present case, the lesion excised by ESD was diagnosed as eCuraA (differentiated type‐dominant adenocarcinoma, pT1a, UL1, ≤ 3 cm) on pathological examination and follow‐up with annual endoscopy according to the guidelines. However, 16 months later, local recurrence with the submucosal tumor‐like appearance, lymph node metastasis, and multiple liver metastases were observed. After the diagnosis of metastatic recurrence was made, an expert pathologist reviewed the ESD specimen with deeper cuts and immunochemical staining such as D2‐40. No non‐curative factors such as submucosal invasion or lymphovascular invasion were observed. A multicenter prospective cohort study of long‐term survival after endoscopic resection for early gastric cancer showed that local recurrence and metastatic recurrence were observed in 0.15% (10/6855) and 0.06% (4/6855) of patients with eCuraA, respectively.^4^ Although infrequent, there exists the potential for local or metastatic recurrence in patients who achieved eCuraA.

Kawabata et al. reported a case of local recurrence after curative ESD of intramucosal differentiated early gastric cancer with a maximum diameter of 30 mm.^5^ Most local recurrent gastric cancers detected during a close follow‐up are intramucosal carcinomas. Notably, it has been documented that ESD serves as a safe and effective treatment for local recurrent gastric cancer subsequent to endoscopic resection, resulting in favorable long‐term outcomes.^6^ Hanaoka et al. reported a case of lymph node and liver metastasis after curative ESD in a patient with pathologically mixed‐type intramucosal gastric cancer.^7^ A review of PubMed revealed only seven case reports of local recurrence, lymph node metastasis, or liver metastases in patients who achieved eCuraA (Table S1); thus, this is the first report of local recurrence with the submucosal tumor‐like appearance and distant metastasis.

The causes of local and metastatic recurrence after ESD are different. Local recurrence is generally caused by tumor remnants resulting from incomplete resection. Lee et al. reported that risk factors associated with tumor remnants included long procedure times, lesions located in the upper third of the stomach, and margins less than 1 mm.^8^ In addition, the presence of undifferentiated gastric cancer components and ulcerative findings have been reported as risk factors for lymph node recurrence.^9^, ^10^ In the case of Hanaoka et al., pathological examination revealed no ulcerative findings; however, poorly differentiated components were observed, which was a risk factor for lymph node recurrence.^7^ In the present case, we found no tumor at the resection margin and no evidence of an undifferentiated gastric cancer component. However, due to the extensive ulcer scarring observed in the submucosal layer, there may have been a residual latent tumor in the vertical margin, or non‐curative factors such as lymphovascular invasion may have remained undetected.

In conclusion, we encountered a rare case of local recurrence with the submucosal tumor‐like appearance, lymph node metastasis, and liver metastases in a patient who had achieved eCuraA. While it is true that the majority of patients in whom eCuraA is achieved do not experience recurrence and have a favorable long‐term prognosis, in cases of early gastric cancer with UL1 or long procedure times, the presence of non‐curative factors such as lymphovascular invasion should be re‐evaluated and careful follow‐up is necessary to consider the possibility of metastatic recurrence despite curative resection.

## CONFLICT OF INTEREST STATEMENT

None.

## Supporting information

Table S1 Seven case reports of recurrence despite eCuraA ESD resection.

References in Supplemental Table 1.

## References

[1] Gotoda T , Yanagisawa A , Sasako M *et al.* Incidence of lymph node metastasis from early gastric cancer: Estimation with a large number of cases at two large centers. Gastric Cancer 2000; 3: 219–225. 11984739 10.1007/pl00011720

[2] Hasuike N , Ono H , Boku N *et al.* A non‐randomized confirmatory trial of an expanded indication for endoscopic submucosal dissection for intestinal‐type gastric cancer (ct1a): The Japan Clinical Oncology Group study (jcog0607). Gastric Cancer 2018; 21: 114–123. 28224238 10.1007/s10120-017-0704-y

[3] Japanese Gastric Cancer Association . Japanese gastric cancer treatment guidelines 2021 (6th edition). Gastric Cancer 2023; 26: 1–25. 36342574 10.1007/s10120-022-01331-8 PMC9813208

[4] Suzuki H , Ono H , Hirasawa T *et al.* Long‐term survival after endoscopic resection for gastric cancer: Real‐world evidence from a multicenter prospective cohort. Clin Gastroenterol Hepatol 2023; 21: 307–318.e2. 35948182 10.1016/j.cgh.2022.07.029

[5] Kawabata H , Kawakatsu Y , Yamaguchi K *et al.* A rare case of local recurrence following curative endoscopic submucosal dissection of intramucosal differentiated‐type gastric cancer. Gastroenterology Res 2019; 12: 103–106. 31019622 10.14740/gr1159 PMC6469905

[6] Sekiguchi M , Suzuki H , Oda I *et al.* Favorable long‐term outcomes of endoscopic submucosal dissection for locally recurrent early gastric cancer after endoscopic resection. Endoscopy 2013; 45: 708–713. 23918620 10.1055/s-0033-1344332

[7] Hanaoka N , Tanabe S , Higuchi K *et al.* A rare case of histologically mixed‐type intramucosal gastric cancer accompanied by nodal recurrence and liver metastasis after endoscopic submucosal dissection. Gastrointest Endosc 2009; 69: 588–590. 19136109 10.1016/j.gie.2008.05.030

[8] Lee JY , Cho KB , Kim ES *et al.* Risk factors for local recurrence after en bloc endoscopic submucosal dissection for early gastric cancer. World J Gastrointest Endosc 2016; 8: 330–337. 27076871 10.4253/wjge.v8.i7.330 PMC4823671

[9] Sekiguchi M , Kushima R , Oda I *et al.* Clinical significance of a papillary adenocarcinoma component in early gastric cancer: A single‐center retrospective analysis of 628 surgically resected early gastric cancers. J Gastroenterol 2015; 50: 424–434. 25142800 10.1007/s00535-014-0991-6

[10] Lee JH , Choi IJ , Han HS *et al.* Risk of lymph node metastasis in differentiated type mucosal early gastric cancer mixed with minor undifferentiated type histology. Ann Surg Oncol 2015; 22: 1813–1839. 25344305 10.1245/s10434-014-4167-7

