# Supplementary material for: A case of early gastric cancer with metastatic recurrence following curative endoscopic submucosal dissection
Source: DEN Open. 2023 Dec 28;4(1):e326. doi: 10.1002/deo2.326 (PMC10753630; doi:10.1002/deo2.326)
Supplement: Supplementary file 1 — Table S1 Seven case reports of recurrence despite eCuraA ESD resection. [file DEO2-4-e326-s002.docx]

**Supplemental Table 1.**

| **Case** | **Year** | **Author** | **Age/sex** | **Differentiation** | **Size**  **(mm)** | **UL** | **Recurrence** | **Duration to recurrence** | |
| --- | --- | --- | --- | --- | --- | --- | --- | --- | --- |
| 1 | 2009 | Hanaoka et al.^1^ | 60/male | Differentiated type-dominant | 55 | 0 | Lymph node  Liver | 14 months | |
| 2 | 2014 | Kim et al.^2^ | 72/male | Differentiated type | 20 | 0 | Lymph node | 12 months | |
| 3 | 2015 | Fujii et al.^3^ | 70/male | Differentiated type | 22 | 1 | Lymph node | 17 months | |
| 4 | 2019 | Kawabata et al.^4^ | 78/male | Differentiated type | 30 | 0 | Local | 24 months | |
| 5 | 2021 | Kamiya et al.^5^ | 76/female | Differentiated type | 30 | 0 | Lymph node | 48 months | |
| 6 | 2022 | Shin et al.^6^ | 57/female | Differentiated type | 20 | 0 | Lymph node | 60 months | |
| 7 | 2022 | Iimori et al.^7^ | 73/male | Differentiated type-dominant | 54 | 0 | Lymph node | 132 months | |
| 8 | 2023 | Our case | 70/male | Differentiated type | 3 | 1 | Local  Lymph node  Liver | 16 months | |
| eCuraA, endoscopic curability A; ESD, endoscopic submucosal dissection; UL, ulcerative finding | | | | | | | | |  |

**References in Supplemental Table 1**

1 Hanaoka N, Tanabe S, Higuchi K *et al.* A rare case of histologically mixed-type intramucosal gastric cancer accompanied by nodal recurrence and liver metastasis after endoscopic submucosal dissection. *Gastrointest Endosc* 2009; **69**: 588-90.

2 Kim DJ, Kim W. A case of single lymph node metastasis near the common hepatic artery following a curative endoscopic resection for gastric mucosal cancer. *Gastric Cancer* 2014; **17**: 387-91.

3 Fujii H, Ishii E, Tochitani S *et al.* Lymph node metastasis after endoscopic submucosal dissection of a differentiated gastric cancer confined to the mucosa with an ulcer smaller than 30 mm. *Dig Endosc* 2015; **27**: 159-61.

4 Kawabata H, Kawakatsu Y, Yamaguchi K *et al.* A rare case of local recurrence following curative endoscopic submucosal dissection of intramucosal differentiated-type gastric cancer. *Gastroenterology Res* 2019; **12**: 103-6.

5 Kamiya A, Katai H, Ishizu K *et al.* Recurrence after esd curative resection for early gastric cancer. *Surg Case Rep* 2021; **7**: 5.

6 Shin HW, Park JY, Bae HI, Park KB, Kwon OK. Regional lymph node recurrence without intragastric lesions after curative endoscopic resection of early gastric cancer meeting the absolute indications of endoscopic resection: A case report. *Medicine (Baltimore)* 2022; **101**: e29417.

7 Iimori K, Tanaka Y, Fujii S, Shibuya S, Kusaka T. Late recurrence of early gastric cancer 11 years after curative endoscopic submucosal dissection. *Int Cancer Conf J* 2022; **11**: 62-6.
